# Re-designing a rapid response system: effect on staff experiences and perceptions of rapid response team calls

**DOI:** 10.1186/s12913-020-05260-z

**Published:** 2020-05-29

**Authors:** Richard Chalwin, Lynne Giles, Amy Salter, Karoline Kapitola, Jonathan Karnon

**Affiliations:** 1 School of Public Health, Faculty of Health and Medical Sciences, University of Adelaide, Adelaide, SA 5005 Australia; 2 Rapid Response System, Lyell McEwin Hospital, Elizabeth Vale, SA 5112 Australia; 3 College of Medicine and Public Health, Flinders University, Bedford Park, SA 5042 Australia

**Keywords:** Hospital rapid response team, Quality improvement, Interdisciplinary communication

## Abstract

**Background:**

Rapid Response Team (RRT) calls are clinical crises. Clinical and time pressures can hinder effective liaison between staff who call the RRT (‘users’) and those responding as part of the RRT (‘members’). Non-technical skills (NTS) training has been shown to improve communication and cooperation but requires time and financial resources that may not be available in acute care hospitals. Rapid Response System (RRS) re-design, aiming to promote use of NTS, may provide an alternative approach to improving interactions within RRTs and between members and users.

**Methods:**

Re-design of an existing mature RRS was undertaken in a tertiary, metropolitan hospital incorporating the addition of: 1) regular RRT meetings 2) RRT role badges and 3) a structured member-to-user patient care responsibility “hand-off” process. To compare experiences and perceptions of calls, users and members were surveyed pre and post re-design.

**Results:**

Post re-design there were improvements in members’ understanding of RRT roles (*P* = 0.03) and responsibilities (*P* < 0.01), and recollection of introducing themselves to users (*P* = 0.02). For users, after the re-design, there were improvements in identification of the RRT leader (*P* < 0.01), and in the development of clinical plans for patients remaining on the ward at the end of an RRT call (*P* < 0.01). However, post-re-design, fewer users agreed that the structured hand-off was useful or that they should be involved in the process. Both members and users reported fewer experiences of conflict at RRT calls post-re-design (both *P* < 0.01).

**Conclusion:**

The RRS re-design yielded improvements in interactions between members in RRTs and between RRT members and users. However, some unintended consequences arose, particularly around user satisfaction with the structured hand-off. These findings suggest that refinement and improvement of the RRS is possible, but should be an ongoing iterative effort, ideally supported by staff training.

**Trial registration:**

NCT01551160. Registered: 12th March 2012.

## Background

The Rapid Response System (RRS) is an integral patient safety mechanism within acute hospitals. It incorporates the afferent limb: a recognition and alert process for clinical deterioration, and the efferent limb: a team-based response to achieve appropriate and timely patient management [1].

Staff for the afferent limb are typically ward clinicians under whose care patients are admitted. The efferent limb Rapid Response Team (RRT) comprises specialised clinicians from acute areas such as the Intensive Care Unit (ICU) [2]. Optimal functioning of the RRS depends on collegial liaison between staff from these two components – those that call the RRT (‘users’) and those rostered to the RRT (‘members’).

The clinical and time stressors of RRT calls can threaten the working relationship between users and members. An impaired interface between RRT members and users may hinder successful resolution of RRT calls [3, 4]. Unaddressed clinical deterioration and/or other patient wellbeing concerns may result in repeat activation of the RRT by the afferent limb. This potentially avoidable repeat calling has been associated with increased in-hospital mortality [5].

Key non-technical skills (NTS) domains, such as communication and cooperation, play a significant role during RRT member-user interactions [6, 7]. Effective use of these skills can be improved through delivery of NTS training to acute care clinicians [8–11]. Unfortunately, education programs require considerable time, logistic and financial resources to be effective, and thus are not always feasible to deliver to frontline hospital staff.

Given these constraints, an alternative approach is to incorporate design elements into the RRS that would promote effective communication and cooperation within the RRS without the need for dedicated training [7, 12, 13]. Previous studies with similar objectives have reported modification of individual aspects of the RRS, albeit without detailed investigation of their effects on system performance [6, 14–19]. Therefore, the present study was conducted to describe and assess a multi-faceted re-design of an RRS which aimed to improve the quality of RRT member-member and member-user communication and cooperation.

## Methods

**Fig. 1:**
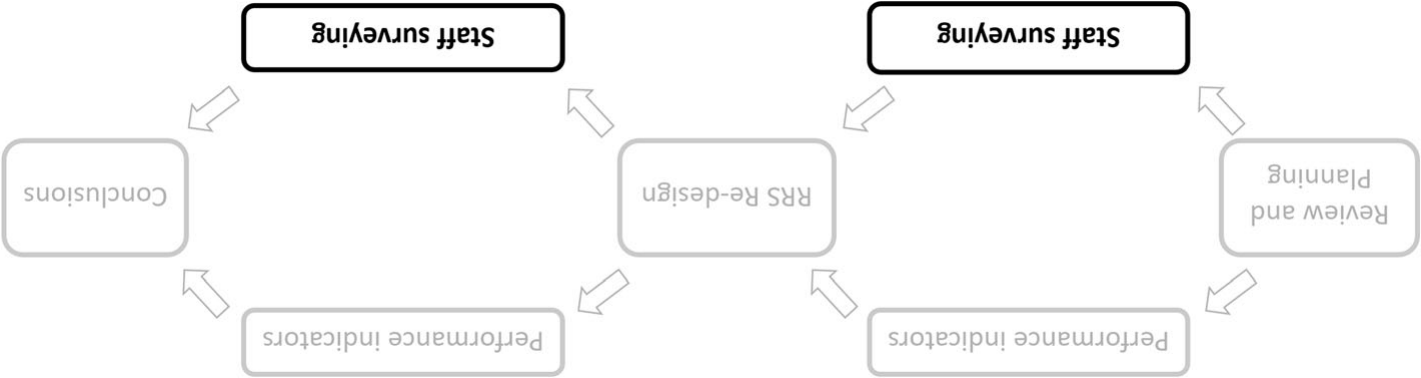
Components of the RRS re-design project. Pre and post re-design hospital staff surveying, as compared in this study, is highlighted. RRS = Rapid Response System

A pre-post survey was conducted as part of the *Impact of Non-Technical Skills on Performance and Effectiveness of a Medical Emergency Team* project (ClinicalTrials.gov: NCT01551160 – a diagram showing the structure of the overall project and the position of this study within it is shown in Fig. 1), comparing clinical staff experiences and perceptions of RRT calls before and after the re-design of a hospital RRS.

Staff at a tertiary, university-affiliated hospital were eligible for inclusion if working in a clinical role during the study. Participants were divided into two groups, RRT members and RRT users.

### The RRS re-design

Incident reports and focus groups conducted at the investigating hospital prior to commencement of the project had highlighted issues around the quality of communication and cooperation during RRT calls, both at the member-user interface and within the RRT.

Insufficient financial and human resources were available at the investigating hospital to deliver an NTS training program for RRS staff. Therefore, a multi-faceted re-design of the existing mature RRS was undertaken instead, incorporating themes from the TeamSTEPPS® program and previously reported RRS improvement initiatives, to promote use of NTS without the need for training [14, 17, 19, 20].

The objectives of the re-design were to encourage a better understanding of roles and responsibilities amongst RRT members, improve identification of those roles to afferent limb staff, and enhance communication both within the RRT and at the interface between team members and users.

The re-design incorporated three components:

1. Regular RRT meetings
2. Badges identifying RRT members’ roles
3. A structured “hand-off” procedure from RRT members to users for patients remaining on the ward at the end of a call

**Fig. 2:**
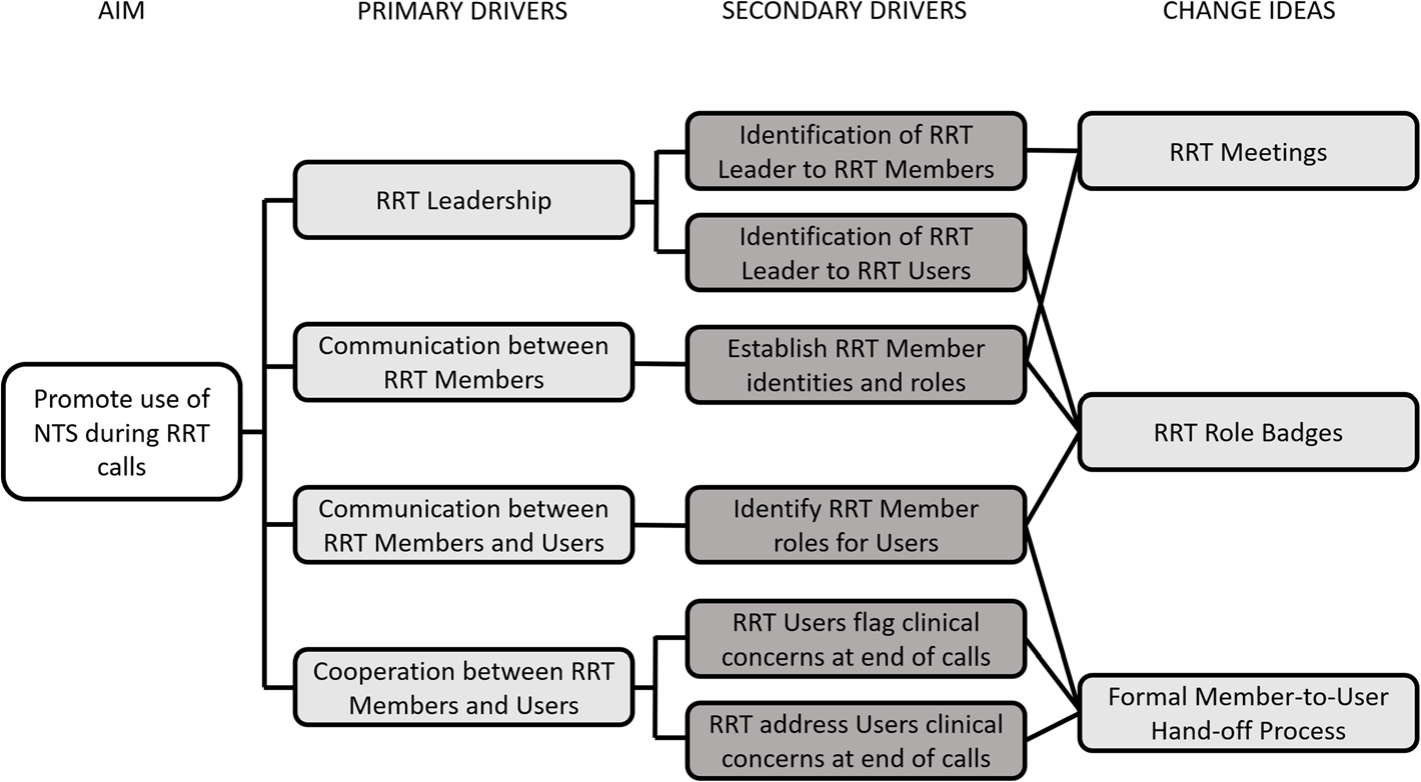
Driver Diagram depicting key drivers and components of the re-design. NTS = Non-Technical Skills, RRT = Rapid Response Team

The relationship of the primary and secondary drivers of these three re-design components are presented in Fig. 2.

#### Regular RRT meetings

The shift-by-shift changeover in RRT staffing was identified as a possible barrier to efficiency [2, 6, 7], since time spent at calls establishing RRT members’ roles and capabilities may delay assessment and resuscitation of patients. Therefore, regular “ice breaker” meetings for RRT staff were implemented [17].

Meetings for RRT staff were scheduled twice daily, to coincide with staff changeovers between day and night shifts, so each team could convene before attending their first call. These meetings, typically lasting around 5 minutes, permitted members’ introductions, and establishment of roles and initial responsibilities when attending calls, especially those of the team leader (see Additional file 1) [6, 7, 17, 20].

#### Team role badges

Feedback from ward staff prior to the re-design suggested that RRT users frequently had difficulty ascertaining RRT membership and roles amongst clinical staff present at calls, with the team leader position particularly challenging to identify. Therefore, RRT role badges were included as part of the re-design to convey member designations to users (Team Leader – usually an ICU resident, RRT Nurse, Medicine Resident, Intern and Hospital Manager) [18].

Badges were distributed during the regular RRT meetings, with members required to wear them conspicuously during calls to ensure that RRT users and other staff could easily identify each member of the team, and their roles, at calls.

#### RRT members-to-users” hand-off” procedure

Prior to an RRT call, each patient’s ward team have responsibility for leading care and clinical decision-making. During a call, this authority is temporarily adopted by the RRT to expedite management of the clinical crisis. However, if the patient is to remain on the ward at the end of their call, this clinical responsibility must be re-assumed by ward staff. Successful completion of the RRT call requires that this transfer of care is not only acknowledged by those on both sides of the member-user interface but is also appropriate. Most importantly, this needs careful consideration regarding whether the patient’s ongoing management needs can be safely and effectively delivered by that ward team [19, 20].

Staff feedback prior to the redesign, and our previous research [3], suggested that unresolved clinical concern at the end of calls was common, resulting in staff unease and, hence, repeat RRT calls. Ensuring resolution of RRT user concern is important as up to 18% of calls prior to the re-design were for the “worried” criterion rather than a predefined physiological trigger [5].

Therefore, a structured verbal and written“ hand-off” protocol was enacted when patients were to remain on their ward after a RRT call. (see Additional file 2). This included the requirement of a signature from a representative of the ward team re-assuming care responsibility to permit stand-down of the RRT, with the intention of encouraging users to voice any ongoing or unresolved clinical concerns before the RRT departed from the call.

### Study phases and survey instrument

The Phase 1 (pre) survey was carried out, following which the RRS re-design, described above, was implemented. One year later, the Phase 2 (post) survey was conducted.

For all survey questions, respondents were asked to recall their experiences and perceptions over the previous 12 months. Therefore, responses for each phase refer to the year preceding the completion of the survey instrument.

Two questionnaires were used: one for RRT members, the other for RRT users, relating to experiences of RRT calls and opinions on the member-user interface (see Additional files 3 and 4). Each group completed a different survey instrument, but the same questionnaire (within group) was repeated in Phase 1 and Phase 2 of the study.

### Data analysis

The effect of the re-design on experiences and perceptions was assessed by comparing Phase 1 and Phase 2 responses. No personal identifiers were collected in the questionnaires to ensure anonymity, so it was not possible to ascertain whether respondents had contributed data to both study phases. As a result, all quantitative data were considered unpaired.

For respondent characteristics, categorical variables are presented as frequencies and percentages and continuous variables are summarised with medians with interquartile ranges (IQRs). Between phase comparisons were conducted by Chi-square tests of association for categorical variables and Mann-Whitney U-tests for continuous variables.

For questionnaire items with Likert scale responses, data were re-coded into binary variables (strongly agree or agree, all other responses). Comparisons of the proportion of agree responses between the study phases for each question were assessed by Z-tests, and results reported as differences in proportions for Phase 2 – Phase 1 (δ2−1), with 95% confidence intervals (95% CI).

Statistical analyses were conducted with SPSS (IBM Corp. Released 2017. IBM SPSS Statistics for Windows, Version 25.0. Armonk, NY: IBM Corp). A *P* value of 0.05 or less was considered statistically significant. No correction for multiple comparisons was made due to the exploratory nature of the study.

Free-text comments from the Phase 2 questionnaire were reviewed and coded if they referred to the RRS re-design. Comments were further categorised into positive (e.g. reporting improvements from the re-design), negative (e.g. identifying problems with the re-design) or suggestions for refinement or improvement. These results are summarised as frequencies.

## Results

### RRT members

There were 79 respondents in Phase 1 and 61 in Phase 2. RRT member roles were similarly represented in each phase except for internal medicine trainees (21 of 79 (26.6%) in Phase 1 vs 4 of 61 (6.6%) in Phase 2, *P* = 0.06). The median number of years of experience as an RRT member was 3 years [IQR 1–6] for Phase 1 respondents versus 2 years [IQR 0.69–5.75] in Phase 2 (*P* = 0.80).

A summary of all RRT members’ questionnaire responses, showing comparisons between Phase 2 and Phase 1, is provided in Table 1. Relative to Phase 1, there was a higher proportion of agree responses in Phase 2 regarding whether the RRT members introduced themselves to users (δ_2–1_ 0.19 [95%CI 0.03–0.36] *P* = 0.02), and understood other team members’ roles (δ_2–1_ 0.12 [95%CI 0.01–0.24] *P* = 0.03) and responsibilities (δ_2–1_ 0.22 [95%CI 0.09–0.34] *P* < 0.01).

**Table 1:** Members’ experiences and perceptions of RRT calls

	Phase 1 N (%) of agree responses	Phase 2 N (%) of agree responses	Differences in Proportions [95%CI]	*P* Value
RRT members introduce themselves to ward staff	34 (43.0%)	38 (62.3%)	0.19 [0.03 – 0.36]	0.02
It is obvious who is the Team Leader at RRT calls	53 (67.1%)	47 (77.0%)	0.10 [-0.05 – 0.25]	0.20
I understand my role as part of the RRT	69 (87.3%)	59 (96.7%)	0.09 [0.00 – 0.19]	0.05
I understand my responsibilities as part of the RRT	74 (93.7%)	60 (98.4%)	0.05 [-0.02 – 0.11]	0.17
The Team Leader delegates roles appropriately	57 (72.2%)	51 (83.6%)	0.11 [-0.03 – 0.25]	0.11
I understand the roles of other members of the RRT	64 (81.0%)	57 (93.4%)	0.12 [0.01 – 0.24]	0.03
I understand the responsibilities of other members of the RRT	58 (73.4%)	58 (95.1%)	0.22 [0.09 – 0.34]	<0.01
The RRT team always receives a handover from the ward team	27 (34.2%)	31 (50.8%)	0.17 [0.00 – 0.33]	0.05
Other members of the RRT listen to and address my queries and concerns	61 (77.2%)	54 (88.5%)	0.11 [-0.1 – 0.24]	0.08
The RRT involves ward staff in development of the clinical plan	62 (78.5%)	53 (86.9%)	0.08 [-0.04 – 0.21]	0.20
The RRT communicates well with other staff	61 (77.2%)	50 (82.0%)	0.05 [-0.09 – 0.18]	0.49
Ward staff who call the RRT are reluctant to be involved during calls	18 (22.8%)	12 (19.7%)	-0.03 [-0.17 – 0.11]	0.66
Attending teams are reluctant to be involved during calls on their patients	17 (21.5%)	16 (26.2%)	0.05 [-0.09 – 0.19]	0.51
I have witnessed conflicts during RRT calls	32 (40.5%)	9 (14.8%)	-0.26 [-0.41 – -0.11]	<0.01
The RRT should handover to ward staff before leaving	76 (96.2%)	55 (90.2%)	-0.06 [-0.14 – 0.02]	0.15
The RRT should not leave until they have an agreed plan with ward staff	75 (94.9%)	53 (86.9%)	-0.08 [-0.17 – 0.01]	0.09
Communication skills are important during RRT calls	79 (100%)	60 (98.4%)	-0.02 [-0.04 – 0.01]	0.25
The RRT works well together	68 (86.1%)	53 (86.9%)	0.01 [-0.11 – 0.12]	0.89

*RRT* Rapid Response Team

Fewer respondents in Phase 2 had witnessed conflicts between staff at RRT calls over the previous year than respondents at Phase 1 (δ_2–1_-0.26 [95%CI -0.41 – -0.11] *P* < 0.01).

For all other questions, the differences in proportions of participants who agreed or strongly agreed (versus not) were not statistically significant between study phases.

### RRT users

There were 297 RRT user respondents in Phase 1 and 302 respondents in Phase 2. RRT user clinical disciplines (e.g. doctor, nurse, allied health clinician) were similarly represented in each phase (*P* = 0.11). The number of years of clinical practice reported by participants was also similar in the two phases.

Similar proportions of respondents had called an RRT in the 12 months prior to each survey (74.4% in Phase 1 vs 77.2% in Phase 2, *P* = 0.57), but more respondents had been directly involved in RRT calls prior to Phase 2 than Phase 1 (86.1% vs 77.1%, *P* = 0.02).

As detailed in Table 2, a higher proportion of respondents in Phase 2 agreed that the RRT leader’s identity was obvious to users (δ_2–1_ 0.21 [95%CI 0.12–0.29] *P* < 0.01) and felt more confident speaking up during RRT calls (δ_2–1_ 0.09 [95%CI 0.01–0.17] *P* = 0.03), relative to respondents in Phase 1.

**Table 2:** Users’ experiences and perceptions of RRT calls

	Phase 1 N (%) of agree responses	Phase 2 N (%) of agree responses	Differences in Proportions [95%CI]	*P* Value
RRT members introduce themselves to ward staff	70 (27.2%)	89 (32.1%)	0.05 [-0.03 – 0.13]	0.22
It is obvious who is the Team Leader at RRT calls	87 (33.9%)	151 (54.5%)	0.21 [0.12 – 0.29]	<0.01
The RRT invites me to state the reason for calling	219 (86.2%)	244 (89.4%)	0.03 [-0.02 – 0.09]	0.27
The RRT acknowledge my rationale for calling	152 (59.8%)	185 (67.8%)	0.08 [0.00 – 0.16]	0.06
The RRT team involve me in patient care during the call	161 (63.1%)	193 (70.2%)	0.07 [-0.01 – 0.15]	0.09
I feel confident speaking to the RRT during calls	166 (65.4%)	204 (74.2%)	0.09 [0.01 – 0.17]	0.03
The RRT communicates well with other staff	157 (63.1%)	191 (70.0%)	0.07 [-0.01 – 0.15]	0.09
I have witnessed conflicts during RRT calls	77 (30.7%)	45 (16.6%)	-0.14 [-0.21 – -0.07]	<0.01
When the patient remains on the ward there is a patient care plan	152 (60.1%)	186 (70.5%)	0.10 [0.02 – 0.19]	<0.01
The RRT team works together to develop a plan for the patient	164 (65.3%)	208 (76.8%)	0.11 [0.04 – 0.19]	<0.01
The RRT involves ward staff in development of the clinical plan	96 (37.9%)	147 (53.8%)	0.16 [0.07 – 0.24]	<0.01
The RRT should not leave until ward staff agree with their plan	261 (91.6%)	160 (58.2%)	-0.33 [-0.41 – -0.26]	<0.01
The RRT should document the clinical plan before leaving	275 (96.5%)	201 (72.8%)	-0.24 [-0.30 – -0.18]	<0.01
The RRT should handover to ward staff before leaving	266 (94.3%)	179 (64.6%)	-0.30 [-0.36 – -0.23]	<0.01
I should be able to read and understand the plan	276 (96.8%)	185 (67.0%)	-0.30 [-0.36 – -0.23]	<0.01
I should feel empowered to ask questions about the plan	267 (93.7%)	163 (59.3%)	-0.34 [-0.41 – -0.27]	<0.01
Poor communication results in recurrent RRT calls	233 (82.0%)	43 (15.8%)	-0.66 [-0.75 – -0.58]	<0.01

*RRT* Rapid Response Team

Furthermore, a higher proportion of respondents at Phase 2 agreed that the RRT developed a clear clinical plan at calls (δ_2–1_ 0.11 [95%CI 0.04–0.19]), involved ward staff in the formulation of those plans (δ_2–1_ 0.16 [95%CI 0.07–0.24]) and ensured that a plan was in place before leaving patients on wards at the end of calls (δ_2–1_ 0.10 [95%CI 0.02–0.19]), all *P* < 0.01.

Experiences of witnessing conflicts between staff at RRT calls were reported less frequently in Phase 2 than Phase 1 (δ_2–1_ -0.14 [− 0.21 – -0.07] *P* < 0.01).

Relative to Phase 1, fewer respondents in Phase 2 agreed that RRT plans should be documented, that ward staff should be invited to read these plans and that their consent should be sought before team departure (δ_2–1_ -0.24 [95%CI -0.30 – − 0.18], − 0.30 [95%CI -0.36 – − 0.23] and − 0.34 [95%CI -0.41 – − 0.27] respectively, all *P* < 0.01).

The proportion of respondents who re-called the RRT to the same patient decreased from 33.3% in Phase 1 to 27.2% in Phase 2, but the difference was not statistically significant (*P* = 0.09). In both study phases, the two most commonly cited reasons were ongoing breaches of calling criteria and unresolved clinical concern that triggered the initial call.

### Phase 2 qualitative data

#### RRT members

Free-text comments were provided by 25 (41.0%) respondents. A total of 19 comments referred to the RRS re-design or its components.

Regarding the RRT meetings, there were five negative comments (e.g. “some [members] don’t always come”) and one suggestion (“have a board of [RRT] staff names and pictures”). For the team role badges there were two negative comments (“not all staff wear them”) and two suggestions for having “stickers rather than badges”. The handovers had two positive comments (e.g. “the contract [handover] is very good”) and one negative comment (“too much paperwork”).

There were three comments pertaining to the overall RRS re-design. Two specifically cited the re-design as having had a positive effect on the RRS (e.g. “communication skills have improved”), whereas the other reported the opposite (“very little [ward] team involvement”).

#### RRT users

Free-text responses were provided by 56 (18.5%) users, with 48 comments relating to aspects of the RRS re-design.

The RRT role badges received three positive comments (e.g. “badges make [RRT member] identification easier”) and four negative ones (e.g. “team leader does not introduce other [RRT] members”). The handovers had three positive comments (e.g. “they leave everyone on the same page”), eight negative comments (e.g. “feel pressured to accept RRT plan”) and four suggestions for improvement (e.g. “handover directly to patient care nurse”).

Twelve user comments praised existing aspects of the re-design for improving interactions with the RRT (e.g. “better attitude and communication”). However, another 12 comments indicated further room for improvement (e.g. “no appreciation that calling is protocolised”).

## Discussion

### Key findings

This study demonstrated improvements in RRT member and user experiences during calls after implementation of a quality improvement re-design of the RRS aimed to facilitate enhanced communication and cooperation.

In particular, both members and users reported a significant decrease in their perceived incidence of conflicts between staff at RRT calls, and a trend towards fewer reports of users having needed to recall the RRT to the same patient, following the RRS re-design.

Despite these positive findings, some aspects of the re-design were less successful. The configuration of the structured hand-off process especially seems to have been problematic.

### Components of the RRS re-design

#### RRT meetings

Improvements in RRT members’ identification of their team leader and understanding of their own and others’ responsibilities suggest that meetings assisted the RRT to establish individual duties prior to attending calls. It is also plausible that patients benefited from resultant expedited management of deterioration due to RRT role allocations having been established prior to attendance, rather than consuming valuable time during calls [6, 17].

Despite the potential benefits of meetings, there were some logistical hurdles. Nurses’ and doctors’ shift changeovers did not always coincide, meaning that occasionally teams would attend calls with members who had not participated in the most recent meeting. Similarly, when rostered RRT staff were on breaks, their substitutes would respond to calls having not attended a meeting.

#### Member role badges

There was an increase in users’ identification of the RRT leader and members’ recognition of each person’s role within the team. These suggest that the badges helped to convey RRT member roles, thereby reducing users’ perceptions of infrequent RRT member verbal introductions to other staff present at calls.

Benefits in efficiency and effectiveness of the management of simulated patient deterioration have been demonstrated when team leaders are easily identifiable [6, 7, 21–23]. However, the contribution of the badges is reliant on them being worn. One RRT member noted that “not all staff wear them” during calls. Some members may have disliked having their designation prominently displayed or inadvertently misplaced their badges.

#### Structured hand-off

The transition of care is fraught with potential risk [24–26]. Amongst these, the need to ensure continuity of clinical responsibility is essential to prevent omissions of, or delays to, decision-making. Commonly used tools for patient handover prompt communication of clinical detail, but do not necessarily prompt users to consider logistics around the transfer of responsibility between teams [25]. Furthermore, handover often does not mandate acknowledgement, documentation or dissemination of the individual or team taking over responsibility [27, 28].

Data before the redesign showed that almost a fifth of all RRT calls to patients were for staff concern [5]. These patients had an in-hospital mortality rate of just over 8 %, in comparison to a national median of less than 1 % for hospital separations [29], despite the absence of a physiological calling criterion being reached. From this it can be inferred that clinician gestalt and intuition should still be taken seriously, even when observations appear to be within normal ranges.

Therefore, the hand-off component of the re-design was carefully constructed and advertised to RRT members and users to encourage the latter group to escalate their concerns, even to the point of delaying completion of the call until satisfied with clinical outcome for the patient. When the transfer of care was by consensus, the hand-off process ensured clear documentation of the clinical team assuming responsibility for that patient’s care beyond the end of the RRT call.

However, this seems to have been the least successful component of the re-design. User responses indicated that some hand-offs were unsatisfactory, took too long, or that users still felt obliged to accept the RRT’s plan despite having unresolved concerns about patient welfare. This latter aspect suggests that some undesirable practices persisted, contrary to the ethos underpinning the re-design.

Interestingly, in Phase 1, RRT users were overwhelmingly in favour of a (re-designed) structured hand-off process [3]. While users apparently support the concept of a formalised transfer of clinical responsibility [19], some aspects of the process implemented in this study did not appear to meet the needs of Phase 2 respondents. It seems likely that modification of a communication procedure may not, alone, be sufficient and that wider organisational cultural change is needed [25].

There were indications that the re-designed hand-off process led to some improvements during member-user interactions. There was a significant increase in respondent agreement that users were involved in devising clinical plans for patients and that these plans were more thoroughly explained to them by the RRT.

#### Overall

The most striking findings were the proportionally large, and statistically significant, reductions in both users’ and members’ perceptions of inter-personnel conflicts at calls. Given the overarching purpose of the re-design was to optimise liaison and teamwork between users and members, these results reassure that the RRS, as a whole, matured to focus on cooperative patient care.

### Strengths and limitations

To the best of the authors’ knowledge, this is the first study to develop and assess a multi-faceted RRS re-design specifically aiming to improve communication and cooperation without the need for NTS training.

The findings from this study should be interpreted with caveats. First, the study did not collect personal identifiers so data could not be analysed to assess intra-individual change. The incentive to participate afforded by anonymity was viewed as more important than the direct comparison of change within individuals. Second, collapsing of Likert scale variables reduced granular information but enabled analysis by proportions of agreement which was important for reporting and interpretation of findings.

Finally, it is recognised that assessment of the effectiveness of handovers or interventions to modify them have been identified as difficult to clearly elicit [30, 31]. In this study of a multi-faceted quality improvement initiative, pragmatic methodology was employed due to a lack of available resources to conduct comprehensive qualitative data collection. Instead, surrogates of staff satisfaction with interactions during calls, such as perceptions of conflicts or needing to recall the RRT, were included as indicators of the broader effect of the initiative on communication and cooperation amongst members and users. More nuanced insights might have been achieved with qualitative data collected through personal interviews or focus groups and subsequent analyses [32], but this was beyond the scope of the present study.

### Lessons for the future

#### Modifying existing components

The improvement of the investigating hospital’s RRS was always anticipated to be an evolving project, of which the design components implemented in this study were one part. In that regard, the evaluation of the re-design has provided useful information about the overall quality improvement process.

The RRT meetings in Phase 2 were reasonably successful. However, since not all members were always able to attend, where possible, a backup option is required. For instance, RRT rosters populated with personnel names, pictures and roles, could be made accessible through hospital intranet websites for those members unable to attend meetings. If resources allowed, this content could be hosted through a mobile app for ease of access by busy clinicians.

The badges also seem to have met their intended purpose, but they are easily misplaced and relatively expensive to replace. Stickers are logical substitutes that can be cheaply printed in bulk and adhered to clothing.

Stickers could also be created for RRT user roles and, along with RRT member stickers, be kept on RRT trolleys for easy access at calls. It is standard practice in Emergency Department resuscitation rooms that roles of all staff are clearly designated during trauma calls, so this should be easily extrapolated to deteriorating patient cases of the RRS.

The structured hand-off process was less successful than intended. Given users’ perceptions of inconvenience, it may be reasonable to make it conditional rather than mandatory. Some RRT calls for simple, self-limiting problems (e.g. a vaso-vagal episode) could be easily flagged as not requiring detailed acknowledgement of resumption of patient responsibility by ward staff. By reserving the structured hand-off process for more complex cases, the true value in ensuring resolution of users’ clinical concern may be realised.

Furthermore, the hand-off proforma assessed in this study included sections for clinical detail and plans. To prioritise its intended function, the proforma could be streamlined to simply record the acknowledgement by, as well as key contact details for, the specific clinical team taking over responsibility for patient care after RRT completion. This could focus all involved clinicians during the member-to-user communication on the importance of continuity of patient care, and further prevent the need for imminent RRT re-activation [3, 5].

#### Need for training

Re-design of RRS structures and procedures can only achieve so much. Ultimately, a comprehensive initiative to improve RRT member and user communication and cooperation would require dedicated training, reinforced by refresher sessions [6–13]. The NTS required by teams involved in the care of deteriorating patients cannot be assumed or innately acquired. Thus, any RRS quality improvements initiatives should ideally include the provision of a “crisis resource management” multi-disciplinary training programme for all RRT members and users [6–8, 10].

## Conclusions

This study showed that improvements in RRT member-user interactions during RRT calls can be attained through introduction of RRT meetings, designation badges and a structured hand-off process. However, it has also identified some challenges in re-designing the structure and procedures of an RRS and its components. This suggests that refinement and improvement of an RRS is possible, but should be seen as a continuously iterative process and supported by a staff education programme.

## Supplementary information

**Additional file 1.** Rapid Response Team meeting checklist.

**Additional file 2.** Rapid Response Team call handover tool.

**Additional file 3.** Rapid Response Team member survey tool.

**Additional file 4.** Rapid Response Team user survey tool.

## Data Availability

The datasets generated and/or analysed during the current study are not publicly available under the terms and conditions of Ethics Committee approval but are available from the corresponding author on reasonable request.

## Abbreviations

RRS: Rapid Response System
RRT: Rapid Response Team
ICU: Intensive Care Unit
NTS: Non-Technical Skills
