# Supplementary material for: Re-designing a rapid response system: effect on staff experiences and perceptions of rapid response team calls
Source: BMC Health Serv Res. 2020 May 29;20:480. doi: 10.1186/s12913-020-05260-z (PMC7257194; doi:10.1186/s12913-020-05260-z)
Supplement: Supplementary file 1 — Additional file 1. Rapid Response Team meeting checklist. [file 12913_2020_5260_MOESM1_ESM.pdf]

RRT Meeting Checklist

Date: __________________________ Time: ______________________ AM/PM

RRT Role	Present?	Full Name	Badge Worn?	BLS / ALS provider?
ICU Registrar	YES / NO		YES / NO	
Medical Reg	YES / NO		YES / NO	
RRT Nurse	YES / NO		YES / NO	
Intern	YES / NO		YES / NO	
Duty Nurse Coordinator	YES / NO		YES / NO	
Orderly	YES / NO		YES / NO	
