# Supplementary material for: Re-designing a rapid response system: effect on staff experiences and perceptions of rapid response team calls
Source: BMC Health Serv Res. 2020 May 29;20:480. doi: 10.1186/s12913-020-05260-z (PMC7257194; doi:10.1186/s12913-020-05260-z)
Supplement: Supplementary file 2 — Additional file 2. Rapid Response Team call handover tool. [file 12913_2020_5260_MOESM2_ESM.pdf]

RRT Call Handover Tool

RRT Call ID: _______________ Date: _____________ Time: _________ AM/PM

RRT Role	Present	Full Name
ICU Registrar	YES / NO	
Med Registrar	YES / NO	
RRT Nurse	YES / NO	
Ward Doctor	YES / NO	
Ward Nurse	YES / NO	
Other	YES / NO	

Clinical Handover (tick when completed):

Identity	Situation	Background	Assessment	Recommend

Ward Team Read-back (tick when completed):

Read-back of plan	Contingency plan	Resus Status	Happy with plan

Signatures:

_____________________________ _____________________________

RRT Leader Ward Doctor / Nurse
