# Supplementary material for: Re-designing a rapid response system: effect on staff experiences and perceptions of rapid response team calls
Source: BMC Health Serv Res. 2020 May 29;20:480. doi: 10.1186/s12913-020-05260-z (PMC7257194; doi:10.1186/s12913-020-05260-z)
Supplement: Supplementary file 3 — Additional file 3. Rapid Response Team member survey tool. [file 12913_2020_5260_MOESM3_ESM.pdf]

What is your role or designation on the RRT team? (please circle)

ICU Registrar Medical Registrar Intern RRT Nurse Duty Nurse Coordinator

Number of years in clinical practice? ……… Number of years on RRT? …………

Question	Strongly agree	Agree	Neutral	Disagree	Strongly disagree
**Currently, when the RRT team arrives**					
RRT members introduce themselves to ward staff					
It is obvious who is the team leader at RRT calls					
I understand my role as part of the RRT team					
I understand my responsibilities as part of the RRT team					
The Team Leader delegates roles appropriately					
I understand the roles of other members of the RRT call team					
I understand the responsibilities of other members of the RRT team					
The RRT team always receives a handover from the ward team					
**Question**	**Strongly agree**	**Agree**	**Neutral**	**Disagree**	**Strongly disagree**
**Over the past year, my experiences during RRT calls are**					
Other members of the RRT listen to and address my queries and concerns					
The RRT involves ward staff during development of the clinical plan					
The RRT team communicates well with other staff					
Ward staff who call the RRT are reluctant to be involved during calls					
Attending teams are reluctant to be involved during calls on their patients					
I have witnessed conflicts during calls					

Question	Strongly agree	Agree	Neutral	Disagree	Strongly disagree
**At the completion of the RRT call**					
The RRT should handover to ward staff before leaving					
The RRT should not leave until they have an agreed plan with ward staff					
**Question**	**Strongly agree**	**Agree**	**Neutral**	**Disagree**	**Strongly disagree**
**Over the past year, my perceptions are**					
Communication skills are important during RRT calls					
The RRT team works well together					
**Question**	**Strongly agree**	**Agree**	**Neutral**	**Disagree**	**Strongly disagree**
**Please rate the following suggestions to help the RRT team improve performance:**					
Meet at the start of a shift to introduce team members and establish the team leader and other team member roles					
Have a debrief at the conclusion of every RRT call					
Receive a handover from the previous shift RRT team					
Follow-up all RRT calls during a shift before the team goes off duty					
Hold regular hospital-wide audit for all patients attended by RRT					
Participate in team-work and communication skills training					
Participate in simulated RRT scenario training					

Do you have other suggestions that would help the RRT team improve their performance?
