# Supplementary material for: Re-designing a rapid response system: effect on staff experiences and perceptions of rapid response team calls
Source: BMC Health Serv Res. 2020 May 29;20:480. doi: 10.1186/s12913-020-05260-z (PMC7257194; doi:10.1186/s12913-020-05260-z)
Supplement: Supplementary file 4 — Additional file 4. Rapid Response Team user survey tool. [file 12913_2020_5260_MOESM4_ESM.pdf]

Usual Ward or Area _________ Designation or Role ________ No. of years graduated ___

Over the past year, have you called an RRT call (please circle) **YES** **NO**

Over the past year, have you been involved in an RRT call (please circle) **YES** **NO**

If the answer is "NO" to both questions, please start on page 2, otherwise answer all questions

Question	Strongly agree	Agree	Neutral	Disagree	Strongly disagree
**When the RRT team Arrives**					
RRT members introduce themselves to ward staff					
It is obvious who is the Team Leader at RRT calls					
The RRT invites me to state the reason for calling					
The RRT acknowledge my rationale for calling					
**Question**	**Strongly agree**	**Agree**	**Neutral**	**Disagree**	**Strongly disagree**
**During the RRT call**					
The RRT team involve me in patient care during the call					
I feel confident speaking to the RRT during calls					
The RRT communicates well with other staff					
I have witnessed conflicts during RRT calls					
**Question**	**Strongly agree**	**Agree**	**Neutral**	**Disagree**	**Strongly disagree**
**At the completion of the RRT call**					
When the patient remains on the ward there is a patient care plan					
The RRT team works together to develop a plan for the patient					
The RRT involves ward staff in development of the clinical plan					

When patients have remained on the ward after a RRT call, I have had to recall the RRT team back to see the same patient (please circle) **YES** **NO**

If "**YES**" please circle why, and rank from most to least common (1 = most common, 7 least common)

	A	No clear plan for the patient
	B	Resus status or treatment limitation orders were not clarified
	C	The reason for calling the previous RRT was not resolved
	D	The patient still was still meeting standard RRT calling criteria
	E	No contingency plan had been made in case that patient reached RRT criteria again
	F	Home team not informed by RRT / unaware of patient remaining on the ward
	G	Other - please explain ____

**Question**	**Strongly agree**	**Agree**	**Neutral**	**Disagree**	**Strongly disagree**
**General Questions about the RRT team / call**					
The RRT should not leave until ward staff agree with the clinical plan					
The RRT should document the clinical plan before leaving					
The RRT should handover to ward staff before leaving					
I should be able to read and understand the plan					
I should feel empowered to ask questions about the plan					
Poor communication results in recurrent RRT calls					

Question	Strongly agree	Agree	Neutral	Disagree	Strongly disagree
If the RRT team leave without my concerns for the patient being resolved and another RRT is necessary for that patient, I would:					
Call another RRT					
Complain to the RRT team					
Call the home team instead					
Call the bed card consultant instead					
Feel discouraged from calling another RRT on the same patient					
Feel discouraged from calling RRT on other patients					

Do you have any other concerns / comments that would be helpful to improve RRT calls

____

____

____

____

____

____
